# The Emerging Role of Ferroptosis in Pediatric Cancer Biology and Therapy

**DOI:** 10.3390/ijms27188067

**Published:** 2026-09-10

**Authors:** Alessandra Di Paola, Maria Maddalena Marrapodi, Giuseppe Di Feo, Martina Di Martino, Daniela Di Pinto, Lucia Argenziano, Oriana Di Domenico, Francesca Rossi, Elvira Pota

**Affiliations:** 1 Department of Life Science, Health and Health Professions, Link Campus University, 00165 Rome, Italy; a.dipaola@unilink.it; 2 Department of Woman, Child and General and Specialist Surgery, University of Campania “Luigi Vanvitelli”, 80138 Napoli, Italy; mariamaddalena.marrapodi@unicampania.it (M.M.M.); giuseppe.difeo.bio@gmail.com (G.D.F.); martina.dimartino@policliniconapoli.it (M.D.M.); daniela.dipinto@policliniconapoli.it (D.D.P.); lucia.argenziano@unicampania.it (L.A.); didomenico.oriana.odd@gmail.com (O.D.D.); elvira.pota@gmail.com (E.P.)

**Keywords:** ferroptosis, pediatric cancer, lipid peroxidation, therapy resistance, tumor microenvironment

## Abstract

Ferroptosis is a regulated form of cell death caused by iron-dependent membrane lipid peroxidation. Iron metabolism, membrane lipid composition, cellular metabolic pathways, and antioxidant defense systems regulate ferroptosis. Recently, ferroptosis has attracted interest as a target in cancer therapy, in particular cancer cells have developed several biological adaptative mechanisms to evade ferroptosis, thus enhancing tumor progression, metastatic dissemination, stemness, and resistance to conventional therapies. Interestingly, ferroptosis is regulated by the tumor microenvironment, where hypoxia, immune cells, and stromal components can either stimulate or inhibit ferroptotic cell death. It has been demonstrated that targeting ferroptosis, alone or in combination with chemotherapy, radiotherapy and immunotherapy, has anticancer effects in preclinical models and may contribute to avoid treatment resistance. However, the role of ferroptosis in pediatric cancer remains incompletely understood since these kinds of tumors show different developmental, genomic, and metabolic features that may contribute to create different ferroptosis vulnerabilities. Emerging evidence in neuroblastoma, B-cell acute lymphoblastic leukemia, osteosarcoma, and medulloblastoma supports the involvement of ferroptosis-related pathways in tumor biology and treatment response. Preclinical studies also indicate that ferroptosis induction may represent a therapeutic strategy in selected pediatric cancer models, although tumor heterogeneity, drug delivery, and potential toxicity to developing tissues remain important translational challenges. This review summarizes the molecular mechanisms underlining the relationship between ferroptosis and cancer biology, tumor progression, tumor microenvironment, and therapy resistance, with particular interest in its emerging relevance and therapeutic potential in pediatric oncology, while critically considering the current evidence and major challenges for clinical translation.

## 1. Introduction

Regulated cell death (RCD) is a pivotal biological process with a key role in embryonic development, tissue homeostasis, immune regulation, and the removal of damaged or malignant cells [1,2]. Over the past decade, apoptosis has been regarded as the primary form of programmed cell death and has been the main biological mechanism investigated for anticancer therapies [3,4]. However, it has been widely demonstrated that many cancer cells have acquired the ability to evade apoptosis through various adaptive mechanisms—both genetic and metabolic—thus promoting tumor progression, metastasis development, and resistance to anticancer therapies [3,4,5,6,7]. Therefore, increasing efforts have been dedicated for identifying novel regulated cell-death pathways which can be therapeutically exploited to eliminate cancer cells [1,8]. Among these, ferroptosis has emerged as one of the most promising targets for the development of innovative anticancer therapies [4,9].

Ferroptosis was first described by Dixon et al. in 2012 as a distinct form of non-apoptotic iron-dependent cell death characterized by lipid peroxide accumulation [10]. Ferroptosis has emerged as a key regulator of cancer biology, given its specific molecular characteristics and its ability to eliminate malignant cells which are resistant to apoptosis [11]. Particularly, it has been demonstrated that ferroptosis is a biological process tightly regulated by a complex network involving iron metabolism, lipid peroxidation, amino acid metabolism, and antioxidant defense systems [1]. These features make it an attractive therapeutic strategy for cancer treatment [4,11].

Cancer cells are characterized by intense metabolic activity, since they proliferate rapidly and survive under conditions of extreme stress [12,13]. In particular, they have high iron requirements, produce high levels of reactive oxygen species (ROS), and exhibit altered lipid metabolism, thereby contributing, on one hand, to tumor growth, and, on the other, to increased cellular susceptibility to ferroptosis [3,4,7]. Consequently, ferroptosis has attracted considerable attention as a promising therapeutic target to eliminate cancer cells that have developed resistance to conventional anticancer therapies [14,15]. Furthermore, growing evidence highlighted that the pharmacological induction of ferroptosis can enhance the efficacy of chemotherapy, radiotherapy, immunotherapy, and targeted therapies, suggesting its potential role in combination therapeutic approaches [3,4,15].

Although ferroptosis has been extensively studied in adult cancer, emerging evidence shows that this form of programmed cell death also plays an important role in pediatric cancer [16,17]. Pediatric cancers comprise a heterogeneous group of malignancies that differ from adult tumors in terms of their developmental origin, relatively low mutational burden, genomic alterations, and metabolic characteristics [18,19,20]. These distinctive biological features could influence the molecular regulation of ferroptosis and the sensitivity of pediatric cancer cells to ferroptotic cell death. Current evidence supporting the involvement and therapeutic relevance of ferroptosis in pediatric cancer is summarized in Table 1.

Over the past few decades, advances in multimodal cancer treatments have significantly improved survival rates among pediatric cancer patients. However, disease relapse, therapy resistance, and the short- and long-term side effects associated with chemotherapy and radiation therapy continue to represent major clinical challenge [21,22]. Consequently, the discovery of novel therapeutic approaches able to selectively target malignant cells, reducing toxicity to normal tissues, is necessary.

Although research on the role of ferroptosis in pediatric cancer is increasing, the scientific evidence on this topic remains limited and is restricted to certain types of pediatric cancer, such as neuroblastoma, acute lymphoblastic leukemia, osteosarcoma, and medulloblastoma [17,23,24,25]. However, data from existing preclinical studies indicate that ferroptosis may represent a promising therapeutic target to enhance treatment efficacy and to reduce the onset of treatment resistance in pediatric oncology [17,23,26].

**Table 1:** Main primary experimental evidence of ferroptosis in pediatric cancers. The table summarizes tumor and model context, molecular determinants, experimental interventions and comparators, functional evidence supporting ferroptosis, in vivo validation, and the principal limitations of the available studies. Evidence levels distinguish mechanistic studies, preclinical therapeutic studies, and patient-associated correlative findings. PDX, patient-derived xenograft; BMSC, bone marrow mesenchymal stromal cell; CAF-MSC, cancer-associated fibroblast-like mesenchymal stromal cell; GSH, glutathione; GPX4, glutathione peroxidase 4; MUFA, monounsaturated fatty acid; PUFA, polyunsaturated fatty acid; NK, natural killer; B-ALL, B-cell acute lymphoblastic leukemia.

Pediatric Cancer/Subgroup	Model Source & Developmental Context	Molecular Context	Intervention/Comparator	Ferroptosis Evidence & Principal Result	In Vivo Validation	Evidence Level/Main Limitation	Ref.
**MYCN-amplified neuroblastoma**	Pediatric neuroblastoma cell models and PDXs	MYCN amplification; increased iron dependency	System Xc^-^-GSH targeting; ferroptosis inhibition/iron chelation as functional controls	Increased iron uptake and ROS create ferroptosis vulnerability; targeting the System Xc^-^-GSH axis induces ferroptosis and inhibits tumor growth	Yes, PDX	Mechanistic + preclinical therapeutic; clinical validation lacking	[27]
**MYCN-amplified neuroblastoma**	Pediatric-origin neuroblastoma cell models	MYCN-TFRC-iron-GPX4 axis	GPX4 inhibition; MYCN/TFRC modulation vs. controls	MYCN-dependent TFRC upregulation increases labile iron and lipid peroxidation; genetic modulation and ferroptosis rescue support a causal relationship with GPX4 sensitivity	No	Mechanistic; predominantly cell-model evidence	[28]
**MYCN-high/amplified neuroblastoma**	Childhood neuroblastoma cell models and orthotopic model	MYCN-driven cysteine dependency; GSH/GPX4	Cysteine deprivation/pathway targeting vs. controls	Limiting cysteine availability compromises GSH synthesis, increases lipid peroxidation and promotes ferroptosis	Yes, orthotopic model	Mechanistic + preclinical therapeutic; clinical validation lacking	[29]
**High-risk/chemoresistant neuroblastoma**	Patient-derived PDXs and PDX-derived organoids	Ferroptosis-related antioxidant pathways; GPX4	Auranofin ± chemotherapy; ferroptosis-targeting approaches	Ferroptosis induction enhances chemotherapy efficacy; chemotherapy-induced GPX4 upregulation can antagonize GPX4-targeting ferroptosis inducers	Yes, PDX	Preclinical therapeutic; findings require clinical validation	[26]
**High-risk neuroblastoma**	Tumor samples from 27 pediatric patients and experimental models	Ferroptosis-associated metabolic and antioxidant programs	Comparison of high-risk tumor-associated molecular profiles	High-risk tumors show enrichment of ferroptosis-associated metabolic pathways together with increased GSH metabolism and GPX4 expression	—	Patient-associated/correlative; no causal evidence of ferroptosis	[30]
**Neuroblastoma-associated stromal cells**	Patient-derived NB-BMSCs and CAF-MSCs; preclinical model	System Xc^-^-GSH-GPX4 axis	Erastin/RSL3; comparison with normal BMSCs	Neuroblastoma-associated stromal cells show impaired anti-ferroptotic defenses and increased susceptibility to ferroptosis compared with normal BMSCs	Yes, preclinical model	Mechanistic + preclinical; clinical relevance of stromal ferroptosis remains to be established	[31]
**Drug-resistant neuroblastoma**	HTLA-ER resistant cell model of pediatric origin	BMI-1/GSH-dependent antioxidant defense	PTC596-mediated BMI-1 inhibition	BMI-1 inhibition reduces GSH and increases peroxide production and lipid peroxidation, promoting ferroptotic cell death	No	Preclinical, in vitro; single resistant-cell model	[32]
**High-risk neuroblastoma**	Pediatric-derived cell models and xenografts	SCD1-mediated MUFA/PUFA remodeling	Genetic/pharmacological SCD1 inhibition; arachidonic acid supplementation	SCD1-dependent MUFA synthesis promotes ferroptosis resistance; SCD1 inhibition or lipid remodeling restores ferroptosis susceptibility	Yes, xenograft	Mechanistic + preclinical therapeutic; clinical validation lacking	[33]
**Neuroblastoma**	Pediatric-derived cell models and NK-cell co-culture	ATF4-ULBP1-NKG2D signaling	Erastin ± Liproxstatin-1; NK-cell co-culture	Erastin-induced ferroptosis increases ULBP1 and enhances NK-cell-mediated cytotoxicity; Liproxstatin-1 reduces this effect	No	Mechanistic, in vitro; immune interaction requires in vivo validation	[34]
**SHH medulloblastoma**	Pediatric-derived cell models and orthotopic model	FANCD2-iron-GPX4 axis	FANCD2 silencing ± radiotherapy	FANCD2 deficiency increases Fe^2+^ accumulation and impairs GPX4 activity, promoting ferroptosis and radiosensitivity; combined FANCD2 silencing and radiotherapy reduces tumor growth	Yes, orthotopic model	Mechanistic + preclinical therapeutic; safety and clinical translation remain to be established	[25]
**Cisplatin-resistant osteosarcoma**	Resistant models derived from pediatric-origin MG63 and Saos-2 cells	STAT3/NRF2/GPX4 axis	Erastin/RSL3 or STAT3 inhibition; resistant vs. parental models	Ferroptosis induction or STAT3 inhibition enhances ferroptosis susceptibility and cisplatin sensitivity	No	Mechanistic, in vitro; no in vivo validation	[35]
**CREBBP-altered B-ALL**	Isogenic B-ALL models and pediatric patient-derived model	CREBBP loss-of-function	BCL2 inhibition with venetoclax; CREBBP-deficient vs. proficient controls	CREBBP loss increases sensitivity to BCL2 inhibition; increased lipid peroxidation and Liproxstatin-1 rescue provide functional evidence of ferroptotic cell death	Yes	Mechanistic + preclinical therapeutic; clinical validation lacking	[36]

In this review, we summarize the current knowledge of the molecular mechanisms of ferroptosis and discuss its emerging role in pediatric oncology, particularly in cancer biology, treatment resistance, interactions with the tumor microenvironment, and current therapeutic strategies aimed at exploiting the potential role of ferroptosis in the treatment of pediatric malignancies.

## 2. Molecular Mechanisms of Ferroptosis

### 2.1. Iron Metabolism and ROS Generation

Iron is an essential trace element involved in several physiological processes, including oxygen transport, DNA synthesis and repair, mitochondrial respiration, energy metabolism, and cell growth and death [37]. Therefore, intracellular iron homeostasis is tightly regulated to ensure adequate iron availability while preventing iron-mediated toxicity. Impairment of iron metabolism is related to pathogenesis of several diseases and represents a molecular process which contributes to initiation and execution of ferroptosis [37,38].

Under physiological conditions, iron homeostasis is regulated by a coordinated network that modulates iron intake and storage, its utilization and its export. The main proteins involved in these processes are transferrin (TF), transferrin receptor 1 (TFR1), divalent metal transporter 1 (DMT1), ferritin, nuclear receptor coactivator 4 (NCOA4), hepcidin, and ferroportin-1 (FPN-1) [37].

Circulating ferric iron (Fe^3+^) bound to TF is recognized by TFR1 on cell surface and internalized through receptor-mediated endocytosis. After endosomal acidification, ferric iron is reduced to ferrous iron (Fe^2+^) by six-transmembrane epithelial antigen of the prostate 3 (STEAP3) and subsequently transported into the cytoplasm by DMT1. Once inside cells, iron is utilized in metabolic processes or stored within ferritin, preventing iron-related toxicity. When there is an increased intracellular iron demand, ferritin undergoes selective autophagic degradation through nuclear receptor coactivator 4 (NCOA4)-mediated ferritinophagy pathway, releasing stored iron into the cytoplasmic labile iron pool (LIP). Conversely, excess intracellular iron can be exported through FPN-1, the only known cellular iron exporter [37].

Dysregulation of this mechanism results in intracellular iron accumulation, impaired redox homeostasis, increased ROS production, and oxidative stress, thus promoting cellular susceptibility to ferroptosis [37,38].

Hepcidin is a key peptide hormone produced by the liver involved in the regulation of iron homeostasis. By binding to FPN-1, hepcidin promotes its internalization and lysosomal degradation, inhibiting iron release and determining an increase in intracellular iron concentration. Intracellular iron accumulation contributes to the expansion of the LIP, which consists mainly of metabolically active and redox-active Fe^2+^ and represents one of the major determinants of cellular susceptibility to ferroptosis. Alteration in cellular iron homeostasis results in increases in the LIP contributing to the development of oxidative stress [38]. The ability of iron to readily cycle between the ferrous (Fe^2+^) and ferric (Fe^3+^) oxidation states underlies both its essential physiological functions and its potential cytotoxicity. Particularly, Fe^2+^ promotes Fenton reaction, in which hydrogen peroxide is converted in highly reactive hydroxyl radicals. Consequently, excessive ROS production promotes oxidative stress, leading to damage of nucleic acids, proteins, and membrane lipids [38].

In the context of ferroptosis, iron-dependent ROS accumulation plays a crucial role in initiating lipid peroxidation, which ultimately compromises membrane integrity and triggers ferroptotic cell death. Overall, dysregulation of iron metabolism can determine the increase or decrease in intracellular LIP levels, promoting or suppressing ferroptosis. Intracellular iron is stored as inert iron in ferritin, whereas autophagic degradation of ferritin (ferritinophagy) releases iron stored in ferritin into the LIP; consequently, activation of ferritinophagy increases intracellular iron availability, promotes LIP expansion, and enhances cell susceptibility to ferroptosis, whereas inhibition of NCOA4-mediated ferritinophagy exerts the opposite effect [11].

### 2.2. Lipid Peroxidation and Membrane Damage

As previously discussed, ferroptosis culminates in the iron-dependent peroxidation of membrane polyunsaturated fatty acid-containing phospholipids (PUFA-PLs), causing an increased production and accumulation of lipid peroxides in cell membranes which determines membrane disruption and consequent cell death [11,39].

PUFA-PL synthesis is regulated by acyl-coenzyme A (CoA) synthetase long chain family member 4 (ACSL4) and lysophosphatidylcholine acyltransferase 3 (LPCAT3). Specifically, ACSL4 mediates the conjugation of CoA to PUFAs, thereby generating PUFA-CoA, which are subsequently esterified and incorporated into PLs by LPCAT3, resulting in the formation of PUFA-PLs [29]. PUFA-PLs are particularly susceptible to peroxidation. This process can occur either non-enzymatically—through iron-dependent free-radical reactions such as Fenton chemistry—or enzymatically, primarily through the activity of lipoxygenases (LOXs) and cytochrome P450 oxidoreductase (POR) [11,40,41].

Non-enzymatic lipid peroxidation proceeds through a free-radical chain reaction consisting of three phases: initiation, propagation, and termination. During the initiation phase, the removal of a hydrogen atom from the bis-allylic position from PUFA-PLs leads to the formation of a carbon-centered lipid radical (L·), which rapidly reacts with molecular oxygen, resulting in the formation of a lipid peroxyl radical (LOO·). Subsequently, during the propagation phase, LOO· abstracts a hydrogen atom from an adjacent PUFA-PL, generating a lipid hydroperoxide (LOOH) and a new L·. The newly formed L· can then react with another molecular oxygen, thereby perpetuating the oxidative chain reaction across cellular membrane. Lipid peroxidation terminates when lipid radicals are neutralized by antioxidants or react to form non-radical products [40].

The progressive accumulation of lipid peroxides in cellular membrane contributes to ferroptosis; in particular, it seems that PUFA-phosphatidylethanolamines represents a particularly relevant target of lipid peroxidation. Under ferroptosis-inducing conditions, lipid peroxides appear to initially accumulate in the endoplasmic reticulum and other organelles, and subsequently at the plasma membranes before membrane rupture. Increasing lipid peroxidation alters the biophysical properties of the plasma membrane and increases membrane tension, which activates the mechanosensitive Piezo1 and transient receptor potential (TRP) channels [42,43]. The opening of these channels leads to the influx of Ca^2+^ and Na^+^ and the efflux of K^+^. This ion flux is facilitated by the inactivation of the ATPase-dependent Na^+^/K^+^ pump, most likely due to altered interaction between this protein and peroxidized membrane phospholipids [42,43]. Collectively, these events lead to an alteration in ionic homeostasis and osmotic swelling of the cell, which ultimately results in membrane rupture [42,44,45].

Under physiological conditions, the accumulation of lipid peroxides and the execution of ferroptosis are counteracted by multiple antioxidant defense systems [11,39,40,42]. Among these, the system Xc^-^-GSH-GPX4 axis represents the major protective pathway against ferroptosis [11,39,40,42] and will be discussed in the following section.

### 2.3. Antioxidant Systems Regulating Ferroptosis

As previously mentioned, ferroptosis is normally counteracted in cells by biological antioxidant defense mechanisms that prevent its initiation [11,39,40,42]. There are various defense mechanisms, including the System Xc^-^-GPX4-GSH axis, the FSP1-CoQH_2_ system, the DHODH-CoQH_2_ system, and the GCH1-BH4 system [11,42].

#### 2.3.1. System Xc^-^-GPX4-GSH Axis

The System Xc^-^-GSH-GPX4 axis represents the main defensive pathway against ferroptosis-mediated death in mammalian cells [11,42].

**Figure 1:**
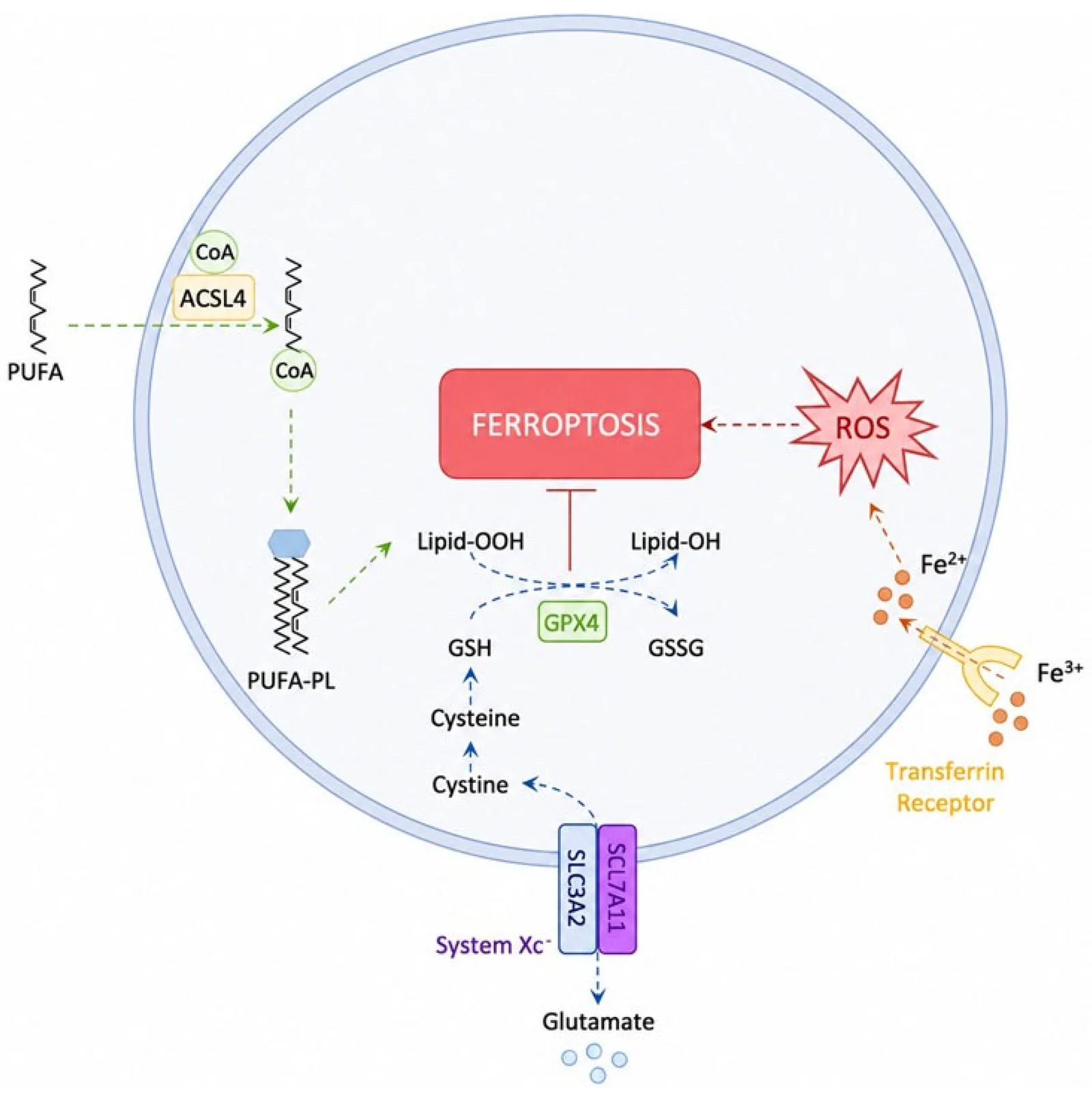
Main molecular mechanisms involved in ferroptosis. Ferroptosis is an iron-dependent form of regulated cell death driven by the accumulation of lipid peroxides. Iron uptake through the transferrin receptor increases the intracellular Fe^2+^ pool and promotes reactive oxygen species (ROS) production. Polyunsaturated fatty acids (PUFAs) are activated by ACSL4 and incorporated into membrane phospholipids (PUFA-PLs), which are particularly susceptible to peroxidation. Ferroptosis is counteracted by the System Xc^-^-GSH-GPX4 axis: cystine uptake induces glutathione (GSH) synthesis, allowing GPX4 to reduce toxic lipid hydroperoxides (Lipid-OOH) to non-toxic lipid alcohols (Lipid-OH). When lipid peroxide accumulation exceeds cellular antioxidant defenses, ferroptotic cell death occurs. Abbreviations: ACSL4, acyl-CoA synthetase long-chain family member 4; CoA, coenzyme A; Fe^2+^, ferrous iron; Fe^3+^, ferric iron; GPX4, glutathione peroxidase 4; GSSG, oxidized glutathione; PUFA-PL, PUFA-containing phospholipid; SLC3A2, solute carrier family 3 member 2; SLC7A11, solute carrier family 7 member 11; System Xc^-^, cystine/glutamate antiporter.

The upstream element of this mechanism is represented by the System Xc^-^ [11]. It is a cystine/glutamate antiporter located at the plasma membrane, constituted by the transporter subunit SLC7A11 and the regulatory subunit SLC3A2 [11,46,47]. This system is responsible for the uptake of extracellular cystine in exchange for intracellular glutamate. Once into the cell, cystine is reduced to cysteine, a precursor for the synthesis of reduced glutathione (GSH). GSH is an essential reducing cofactor for glutathione peroxidase 4 (GPX4). GPX4 is a selenium-dependent enzyme able to directly reduce phospholipid hydroperoxides to their corresponding non-toxic phospholipid alcohols, thus preventing the accumulation of lipid peroxides and consequently the propagation of lipid peroxidation [11,42] (Figure 1).

Therefore, disruption of any component of the System Xc^-^-GSH-GPX4 axis could induce ferroptosis initiation. Particularly, alteration in System Xc^-^ determines a reduction in cysteine availability and consequently decreases GSH levels, thus altering GPX4 activity and enhancing lipid peroxide accumulation. Similarly, genetic deletion or pharmacological inhibition of GPX4 results in uncontrolled lipid peroxidation and leads to ferroptosis [11,42].

#### 2.3.2. FSP1-CoQH_2_ System

In addition to the System Xc^-^-GSH-GPX4 axis, cells also have other GPX4-independent defense mechanisms against ferroptosis. Among them, ferroptosis suppressor protein 1 (FSP1) plays a key role in counteracting ferroptosis [11,48].

FSP1 is a NAD(P)H-dependent oxidoreductase localized on plasma membrane. It reduces ubiquinone, also known as coenzyme Q (CoQ), to its reduced form, ubiquinol (CoQH_2_). CoQH_2_ neutralizes lipid peroxyl radicals, thus inhibiting lipid peroxidation and consequently ferroptosis [11,48,49].

#### 2.3.3. DHODH-CoQH_2_ System

Another GPX4-independent defense mechanism against ferroptosis is represented by the dihydroorotate dehydrogenase (DHODH)-CoQH_2_ system [11,50]. DHODH is an enzyme localized at the inner mitochondrial membrane and takes part in de novo pyrimidine synthesis. Moreover, DHODH participates in ferroptosis defense mechanism by reducing CoQ to its antioxidant form CoQH_2_. CoQH_2_ counteracts lipid peroxidation within the inner mitochondrial membrane, thus protecting cells against ferroptotic cell death [11,50].

Both DHODH and mitochondrial GPX4 play an important role in maintaining mitochondrial membrane integrity. If GPX4 activity is impaired, there is an increase in the production of CoQH_2_, which helps maintain the integrity of the inner mitochondrial membrane by preventing lipid peroxidation [50]. However, if the functions of both GPX4 and DHODH are impaired, there will be a significant increase in mitochondrial lipid peroxidation, leading to the initiation of ferroptosis [50].

Therefore, it is evident that there are compartmentalized defense mechanisms against ferroptosis. In particular, while FSP1 generates CoQH_2_ in non-mitochondrial membranes to prevent ferroptosis, DHODH produces CoQH_2_ in the inner mitochondrial membranes. This underscores the importance of the simultaneous action of both FSP1–CoQH_2_ and DHODH–CoQH_2_ defense systems in preventing ferroptosis [11].

#### 2.3.4. GCH1-BH4 System

The GTP cyclohydrolase 1 (GCH1)-tetrahydrobiopterin (BH4) system represents another defense mechanism which is independent of GPX4 [11,51]. GCH1 takes part in the biosynthesis of BH4, a cofactor involved in several physiological enzymatic reactions [11]. However, BH4 also acts as a potent radical-trapping antioxidant in ferroptosis. It directly neutralizes lipid peroxyl radicals, thus containing the propagation of lipid peroxidation and preventing ferroptosis [51]. Particularly, increased GCH1 activity and consequent BH4 production inhibit ferroptosis initiation. An alteration in this pathway induces lipid peroxidation and ferroptotic cell death [11,51,52].

Moreover, it has been reported that the GCH1-BH4 pathway could regulate ferroptosis through additional mechanisms, including the maintenance of reduced CoQH_2_ pools and the remodeling of membrane phospholipid composition [11].

## 3. Ferroptosis in Cancer Biology

Ferroptosis has an important role in cancer biology, since its regulation can influence tumor development, progression, and response to anticancer therapies [3]. Cancer cells can modulate their susceptibility to ferroptosis through different metabolic and antioxidant mechanisms, making the role of ferroptosis dependent on the biological and molecular characteristics of each tumor [3]. Importantly, pediatric cancers present biological features that distinguish them from adult tumors. Pediatric tumors frequently originate from developing tissues and are generally characterized by a lower mutational burden, with tumorigenesis often associated with alterations in transcriptional activity and chromatin state [18]. These characteristics, in combination with differences in cell of origin and specific oncogenic events, may determine specific metabolic vulnerabilities and influence ferroptosis susceptibility in pediatric cancers [53]. Therefore, investigating ferroptosis in the specific biological and developmental context of pediatric malignancies may provide important insights into cancer progression and the identification of novel therapeutic strategies.

### 3.1. Ferroptosis and Tumor Suppression

It is well known that tumor cells require a continuous supply of nutrients to support their uncontrolled proliferation. Among these nutrients, iron plays a particularly relevant role, since it is involved in different metabolic processes [54,55]. Therefore, cancer cells frequently remodel iron metabolism, by increasing iron uptake and retention, thus ensuring its availability for essential cellular processes, including DNA synthesis, energy metabolism, and mitochondrial function [55]. Iron plays a dual role in cancer, on one hand increased iron availability supports tumor progression, whereas on the other, excessive intracellular iron accumulation can promote ROS production and lipid peroxidation, thereby increasing cellular susceptibility to ferroptosis [55]. Nevertheless, despite their high iron requirements and increased intracellular iron availability, many cancer cells develop adaptive mechanisms that limit ferroptotic cell death. These mechanisms include the regulation of redox homeostasis, lipid metabolism, and iron sequestration, thereby promoting resistance to ferroptosis [55].

Therefore, the ability of cancer cells to evade ferroptotic cell death contributes to tumor development and progression. Conversely, ferroptosis can be considered an intrinsic tumor-suppressive mechanism that promotes the elimination of damaged or transformed cells [9,55].

In pediatric cancers, ferroptosis susceptibility may also be influenced by lineage-specific oncogenic and metabolic programs. Particularly in neuroblastoma, MYCN amplification can profoundly influence cellular susceptibility to ferroptosis. Floros et al. demonstrated that MYCN-amplified neuroblastoma cells increase iron uptake through TFR1, resulting in iron accumulation and increased ROS production, determining a dependence on the System Xc^-^-GSH pathway [27]. Accordingly, targeting this pathway induced ferroptosis and inhibited tumor growth in MYCN-amplified patient-derived xenograft models [27]. Moreover, Lu et al. also showed that MYCN-mediated TFR1 upregulation promoted an increase in the labile iron pool and consequently lipid peroxidation, thus enhancing sensitivity to GPX4 inhibition [28]. Furthermore, Alborzinia et al. identified a strong cysteine dependency in MYCN-amplified childhood neuroblastoma, linking cysteine availability and GSH synthesis to protection against ferroptosis [29]. These findings suggest that ferroptosis susceptibility in pediatric neuroblastoma may be closely related to oncogene-driven metabolic dependencies, highlighting the importance of the specific molecular and developmental context in determining ferroptosis susceptibility [18,20].

It has been reported that several tumor suppressor genes also exert part of their anticancer activity by promoting ferroptotic cell death [3,9]. Among these, TP53 is one of the main genes involved in ferroptosis regulation [3,9,56]. Particularly, p53 can induce ferroptosis by inhibiting SLC7A11 expression, thereby reducing cystine internalization and limiting cellular antioxidant capacity [3,9,57]. This mechanism leads to lipid peroxidation and makes cell more susceptible to ferroptotic cell death. Moreover, p53-mediated ferroptosis also involves the activity of arachidonate 12-lipoxygenase (ALOX12), thus promoting lipid peroxidation [9].

Interestingly, the role of ferroptosis in p53-mediated tumor suppression appears to be partially independent of the canonical tumor-suppressive function of p53; indeed, it has been demonstrated that p53 mutants defective in the induction of apoptosis, senescence and of cell-cycle arrest, but retaining the ability to promote ferroptosis, can still suppress tumor development. Conversely, p53 alterations that impair ferroptosis regulation are associated with the loss of tumor-suppressive activity [58,59,60].

Moreover, ferroptosis can be modulated by other tumor suppressors [3,9]. BRCA1-associated protein 1 (BAP1) is a deubiquitinase involved in ferroptosis regulation, since it is able to epigenetically inhibit SLC7A11 expression. In particular, BAP1 limits SLC7A11 transcription by reducing histone H2A ubiquitination at the SLC7A11 promoter, thus increasing cellular susceptibility to ferroptosis [3,9,61]. Conversely, loss or inactivation of BAP1 does not repress SLC7A11 expression, thus allowing cancer cells to evade ferroptosis and promoting their growth and proliferation. Other studies support the role of BAP1 in ferroptosis induction by demonstrating that its expression inhibits tumor growth via a biological mechanism which is reversed when ferroptosis is inhibited [9,61].

Fumarate hydratase (FH), an enzyme involved in the tricarboxylic acid cycle, also exerts tumor-suppressive activity, by modulating ferroptosis. Cancer cells carrying FH mutations show increased resistance to ferroptosis and continue to proliferate in cystine-deprived conditions [9]. Accordingly, cells expressing wild-type FH are more susceptible to cell death. Therefore, loss of FH function promotes ferroptosis resistance, thus allowing cancer cells to proliferate [9].

Another important mechanism linking ferroptosis to tumor suppression is represented by the KEAP1-NRF2 pathway [9]. Under physiological conditions, KEAP1 induces the proteasomal degradation of NRF2, limiting NRF2-dependent antioxidant responses [3,9,62]. Loss or inactivation of KEAP1 determines NRF2 stabilization and increased expression of anti-ferroptotic proteins, including SLC7A11 and FSP1 [9,63]. Consequently, activation of NRF2-dependent antioxidant defenses promotes ferroptosis cell resistance and contributes to tumor growth and progression [9,62].

Finally, it has been reported that the epigenetic regulator MLL4 is also involved in ferroptosis-mediated tumor suppression [3,9]. MLL4 normally stimulates the expression of lipoxygenases, including ALOX12, thus promoting lipid peroxidation and consequently ferroptotic cell death [9,64]. Conversely, loss of MLL4 is associated with reduced expression of pro-ferroptotic genes (ALOX12, ALOX12B, and ALOXE3) and increased expression of anti-ferroptotic factors (SLC7A11 and GPX4), leading to ferroptosis resistance and tumor development [3,9,64].

Taken together, this evidence suggests that ferroptosis is closely linked to several tumor-suppressive pathways. Although these mechanisms act through distinct molecular processes, they converge on the modulation of cancer cell susceptibility to ferroptotic cell death.

### 3.2. Ferroptosis and Cancer Progression

#### 3.2.1. Ferroptosis and Metabolic Reprogramming

Tumor metabolic reprogramming is a mechanism referred to the metabolic adaptations of cancer cells in response to different environmental and cellular stresses [65]. These adaptations include alteration in glucose, amino acid, and lipid metabolism, allowing cancer cells to meet their increased energy and metabolic demands, thereby supporting tumor growth and progression [65].

Among these metabolic adaptations, reprogramming of glucose metabolism is an important mechanism for regulation of cancer cell susceptibility to ferroptosis [65,66]. Tumor cells frequently increase glycolysis while reduce oxidative phosphorylation, thus limiting excessive ROS production [65,66]. Even though aerobic glycolysis produces less energy than the complete oxidative metabolism of glucose, this metabolic adaptation allows cancer cells prevent excessive oxidative stress, reduce lipid peroxide accumulation, and consequently limit ferroptotic cell death [65,67]. Furthermore, cancer cells can enhance the pentose phosphate pathway (PPP), which contributes to the maintenance of cellular redox homeostasis. In particular, increased PPP activity promotes NADPH production, thereby enhancing cellular antioxidant capacity and limiting the accumulation of lipid peroxides [65]. Consequently, metabolic reprogramming toward the PPP may further protect cancer cells from ferroptosis [65,68].

Amino acid metabolism also plays a key role in regulating cancer cell susceptibility to ferroptosis. Glutamine is a particularly relevant amino acid in the context of ferroptosis since it can exhibit both pro- and anti-ferroptotic properties. Glutamine is converted into glutamate by glutaminase (GLS). Glutamate can be further converted into α-ketoglutarate (α-KG), which enters the tricarboxylic acid (TCA) cycle and supports oxidative phosphorylation, thus increasing ROS production and promoting ferroptosis [65,69]. Accordingly, cancer cells characterized by high TCA cycle activity may exhibit increased glutamine uptake and greater susceptibility to ferroptosis. Conversely, glutamate can also contribute to GSH synthesis, supporting cellular redox homeostasis and protecting cancer cells against ferroptosis [65]. Thus, glutamine metabolism may either promote or suppress ferroptosis.

In MYCN-amplified childhood neuroblastoma, amino acid metabolism represents an important determinant of ferroptosis susceptibility. Interestingly, it has been demonstrated that MYCN induces a high cysteine demand, which is sustained by cystine uptake and transsulfuration. When cystine uptake is limited, cysteine is preferentially used for protein synthesis, thus reducing GSH production and promoting lipid peroxidation and ferroptotic cell death [29]. These findings provide a direct link between oncogene-driven amino acid metabolism and ferroptosis susceptibility in childhood neuroblastoma.

Finally, lipid metabolic reprogramming also influences cancer cell susceptibility to ferroptosis [65,66]. Lipid peroxidation is largely influenced by the balance between PUFAs and monounsaturated fatty acids (MUFAs). In particular, as already discussed, PUFA-PL are highly susceptible to peroxidation and promote ferroptosis; conversely, MUFAs exert the opposite effect by limiting lipid ROS accumulation and reducing PUFA peroxidation, thus inhibiting ferroptosis in cancer cells [65,70]. Interestingly, cancer cells can remodel their lipid composition to limit ferroptosis. In this context, stearoyl-CoA desaturase (SCD), an enzyme involved in MUFA production, is frequently upregulated in tumor cells and contributes to ferroptosis resistance, limiting lipid peroxidation [65,71].

Importantly, recent evidence from pediatric patients further supports the relationship between metabolic reprogramming and ferroptosis in neuroblastoma. Spatial multi-omics analysis of tumor samples from 27 pediatric patients revealed that high-risk neuroblastoma is characterized by enrichment of ferroptosis-associated metabolic pathways, including fatty acid metabolism and ROS signaling. At the same time, high-risk tumors showed increased GSH metabolism and GPX4 expression, suggesting the activation of antioxidant mechanisms that may counteract ferroptosis-associated metabolic stress [30]. These findings provide direct evidence from pediatric tumor samples that metabolic and antioxidant adaptations associated with ferroptosis are relevant features of high-risk neuroblastoma.

#### 3.2.2. Ferroptosis and Cancer Stem Cells

Cancer stem cells (CSCs) represent a subpopulation of cancer cells characterized by self-renewal and differentiation capacity [72,73,74]. CSCs are involved in tumor initiation and progression, tumor growth, and resistance to conventional therapies [74]. Interestingly, it has been reported that CSCs exhibit a peculiar relationship with ferroptosis [73,74]. In particular, CSCs are characterized by an impairment of iron metabolism, with an increased iron internalization and the consequent intracellular iron accumulation [74]. However, although this alteration in iron metabolism could allow for lipid peroxidation, CSCs develop several adaptive mechanisms to evade ferroptotic cell death [74]. Particularly, CSCs maintain low intracellular ROS levels both by reducing oxidative metabolism and promoting antioxidant defenses, including increased activity of the system Xc^-^-GSH axis. Increased SLC7A11 expression promotes cystine uptake and GSH synthesis, thus limiting lipid peroxidation and preventing ferroptosis [74,75]. Moreover, NRF2-dependent defense mechanism can further contribute to ferroptosis resistance [74].

Lipid metabolic reprogramming also contributes to prevent CSCs ferroptotic cell death: increased SCD1 activity promotes MUFA synthesis and lipid droplet accumulation, thus reducing lipid peroxidation [76]. Therefore, despite their high intracellular iron content, CSCs can acquire a ferroptosis-resistant phenotype through coordinated regulation of redox homeostasis and lipid metabolism. These adaptations contribute to CSCs persistence, therapeutic resistance, and tumor recurrence, suggesting that targeting ferroptosis defense mechanisms may represent a promising strategy for the selective elimination of CSCs [74].

#### 3.2.3. Ferroptosis and Metastasis

Cancer cells can proliferate and spread from the primary tumor to other organs, invading them and thus causing metastases [77,78]. The metastatic cascade involves cancer cells undergoing various stages: local invasion, intravasation, survival in the blood or lymphatic circulation, extravasation, and adaptation to distant tissues [77,78,79]. During these stages, cancer cells are exposed to different oxidative and metabolic stresses which can contribute to make them more susceptible to ferroptosis. Therefore, ferroptosis may act as a selective barrier to metastatic progression by removing cancer cells unable to adequately control lipid peroxidation. Conversely, cancer cells able to develop antioxidant defense mechanism, and to modulate iron metabolism and membrane lipid composition can acquire increased ferroptosis resistance and a greater ability to complete the metastatic process [78].

The role of ferroptosis as a selective barrier to metastatic progression is particularly relevant during cancer cell dissemination. Circulating tumor cells (CTCs) are characterized by increased oxidative stress, which lead to ROS production and lipid peroxidation, thus increasing the risk of ferroptotic cell death. Therefore, cancer cell survival in the circulation represents a critical point of the metastatic cascade; particularly, only cancer cells able to develop ferroptosis defense mechanisms can survive and subsequently colonize distant tissues [78].

Interestingly, the blood and lymphatic circulation differentially influence cancer cell susceptibility to ferroptosis. In particular, the lymphatic circulation can provide more favorable conditions for tumor cell survival. In melanoma, the lymphatic environment protects cancer cells against ferroptosis, partly due to its higher content of oleic acid, which reduces lipid peroxidation. This protection increases the ability of melanoma cells to survive subsequent exposure to the blood circulation and promotes metastatic dissemination [78,80].

### 3.3. Ferroptosis Within the Tumor Microenvironment

**Figure 2:**
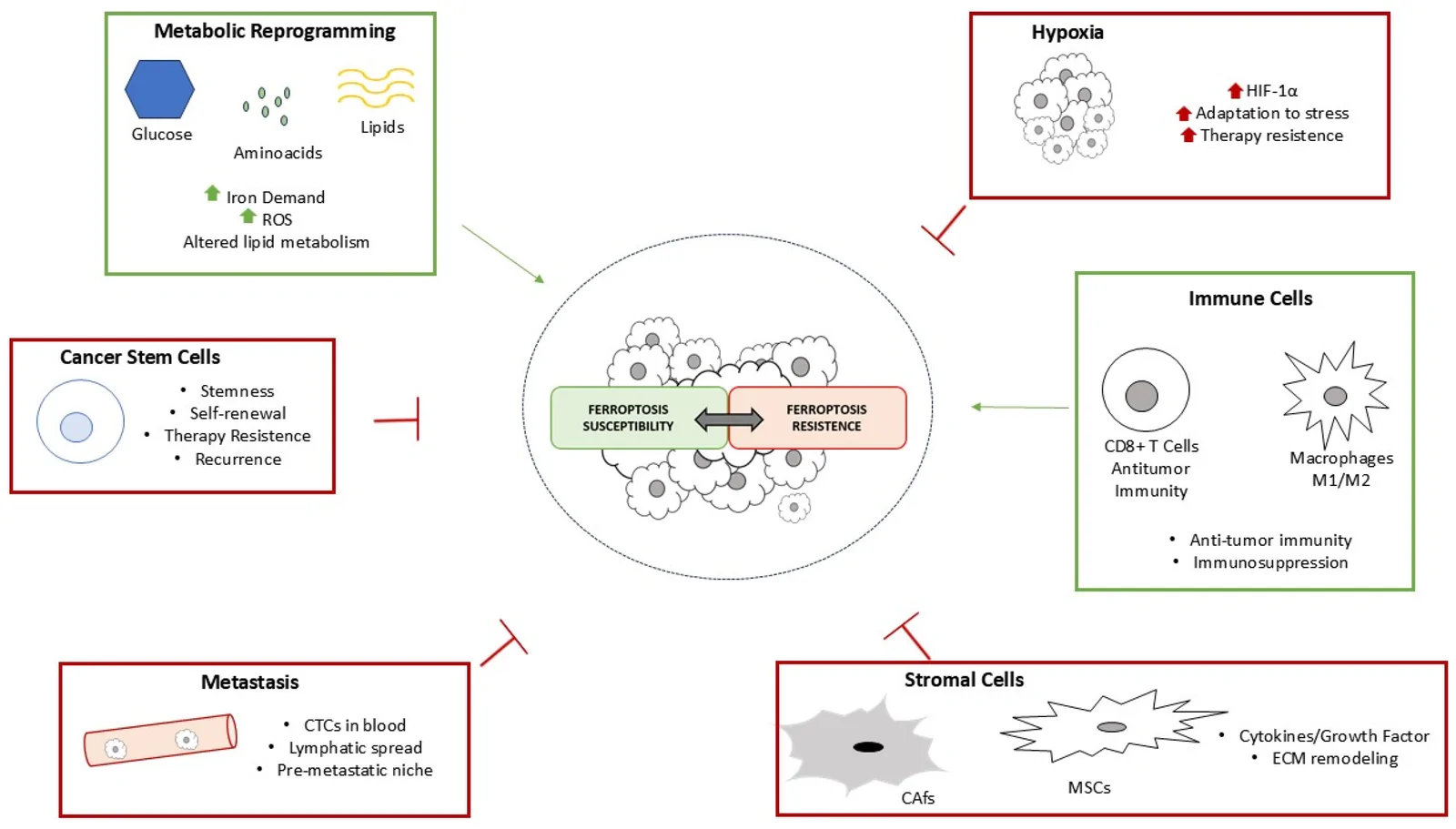
Interplay between ferroptosis, cancer progression, and the tumor microenvironment. Cancer cell susceptibility to ferroptosis is dynamically influenced by metabolic reprogramming, cancer stem cell properties, metastatic dissemination, and tumor microenvironmental factors. Alterations in glucose, amino acid, lipid, and iron metabolism can modify oxidative stress and lipid peroxidation. Cancer stem cells and disseminating tumor cells can develop adaptive mechanisms that promote ferroptosis resistance, thereby supporting therapy resistance, recurrence, and metastatic progression. Within the tumor microenvironment, hypoxia, immune cells, and stromal cells, including cancer-associated fibroblasts (CAFs) and mesenchymal stromal/stem cells (MSCs), can either promote or suppress ferroptosis in a context-dependent manner. The balance between ferroptosis susceptibility and resistance therefore contributes to tumor progression and therapeutic response. Abbreviations: CTCs, circulating tumor cells; ECM, extracellular matrix; HIF-1α, hypoxia-inducible factor 1-alpha; M1/M2, classically/alternatively activated macrophage phenotypes; ROS, reactive oxygen species.

Tumor microenvironment (TME) is a complex biological system constituted by cancer cells and different non-malignant components, including immune cells, stromal cells, extracellular matrix, and several soluble factors [81]. Within the TME, cancer cells are exposed to specific metabolic and environmental conditions which can influence their susceptibility to ferroptosis. Ferroptosis in cancer or non-malignant cells can modify the surrounding microenvironment. Therefore, the relationship between ferroptosis and the TME is bidirectional and can influence tumor progression and therapeutic response [81] (Figure 2).

#### 3.3.1. Hypoxia

Hypoxia is a common feature of solid tumors and represents a key microenvironmental condition influencing tumor biology [82]. It is characterized by inadequate oxygen supply determined by the rapid proliferation of tumor cells and the abnormal organization of tumor vasculature [82]. Under hypoxic conditions, cancer cells develop adaptive mechanisms mainly mediated by hypoxia-inducible factors (HIFs), which induce metabolic reprogramming, angiogenesis, invasion, and resistance to cell death. Recently, it has been reported that hypoxia also modulates cancer cell susceptibility to ferroptosis. Interestingly, hypoxia shows a dual role in modulating ferroptosis: it can either inhibit or promote ferroptotic cell death [82].

In several tumor models, hypoxia is able to induce ferroptosis resistance mainly due to HIF-1α intervention. In particular, HIF-1α participates in different pathways involved in iron metabolism, lipid peroxidation, and antioxidant defenses [82]. For example, hypoxic cancer cells can reduce the intracellular availability of catalytic iron by decreasing TFRC expression and increasing ferritin levels [82]. Moreover, HIF-1α can promote fatty acid uptake and lipid storage by upregulating FABP3 and FABP7, thus limiting the availability of lipids susceptible to peroxidation [82]. Hypoxia can also strengthen antioxidant defenses by increasing the expression or stability of major anti-ferroptotic factors, including SLC7A11 and GPX4 [82]. Together, these mechanisms reduce free iron availability, ROS production, and lipid peroxidation, thus reducing cancer cells susceptibility to ferroptotic cell death.

Nevertheless, hypoxia effect on ferroptosis is not exclusively protective. Indeed, in other tumors HIF signaling can also increase cancer cell susceptibility to ferroptosis [82]. HIF-2α is the main responsible for this mechanism, since it promotes the expression of genes involved in lipid and iron metabolism [82]. For instance, HIF-2α-mediated activation of HILPDA can increase the enrichment of polyunsaturated fatty acids, thereby promoting lipid peroxidation and ferroptosis [82]. HIF-2α has also been associated with increased expression of ferroptosis-related factors, including ACSL4, PTGS2, and CHAC1, in specific cancer models [82]. Therefore, hypoxia exerts a dual and context-dependent effect on ferroptosis, depending on the tumor type, the predominant HIF isoform, and the metabolic characteristics of cancer cells [82].

#### 3.3.2. Immune Interactions

Ferroptosis is closely interconnected with the modulation of the activity of immune cells in TME. Interestingly, this interaction is bidirectional: immune cells can modulate ferroptosis in cancer cells, while ferroptotic cell death can, in turn, influence the recruitment and function of different immune cell populations [83]. Therefore, ferroptosis may either enhance antitumor immunity or contribute to tumor immune evasion [83].

Among immune cells, CD8+ T cells are the key character of the interaction between ferroptosis and antitumor immunity [83]. In particular, activated CD8+ T cells release interferon-γ (IFN-γ), which causes downregulation SLC7A11 expression, thus contributing to lipid peroxidation and, consequently, determining initiation of ferroptosis in cancer cells [83,84]. Therefore, CD8+ T cell-mediated induction of ferroptosis may contribute to the antitumor effects of cancer immunotherapy [83,84].

However, immune cells themselves can also be susceptible to ferroptosis, thus losing their capability to promote cancer cells ferroptotic cell death [83]. In TME, CD8+ T cells can also increase extracellular fatty acids internalization through the scavenger receptor CD36, determining intracellular lipid accumulation and, consequently, lipid peroxidation [83,85,86].

Moreover, ferroptosis is also related to tumor-associated macrophages (TAMs), an important immunological component of the TME [83]. Interestingly, M1- and M2-polarized macrophages show different susceptibility to ferroptosis. M1 macrophages are relatively resistant to ferroptosis since they produce high levels of nitric oxide (NO), which can limit lipid peroxidation; instead, M2 macrophages appear to be more susceptible to ferroptotic cell death [83,87]. Therefore, the selective induction of ferroptosis in M2 macrophages may contribute to restore antitumor immunity [83].

However, the connection between ferroptosis and macrophages could be different based on the specific tumor context. For example, in pancreatic cancer ferroptotic cancer cells can release oncogenic KRASG12D, which stimulates macrophage polarization toward M2 phenotype, thus contributing to tumor progression [83,88].

A pediatric perspective on this interplay has recently emerged in neuroblastoma. In tumor samples from pediatric patients, spatial profiling identified macrophage-enriched regions associated with poorer survival and highlighted macrophage-tumor interactions potentially related to ferroptosis susceptibility [30]. This spatial association suggests that the immune composition of the neuroblastoma microenvironment may contribute to the regulation of ferroptosis, although further functional studies are required to define the underlying mechanisms.

Overall, this evidence highlights that the interaction between ferroptosis and antitumor immunity is highly complex and context-dependent. While ferroptosis of cancer cells can promote immune-mediated tumor elimination, ferroptosis of antitumor immune cells may instead determine immune evasion. Therefore, the biological effects of ferroptosis within the TME depend not only on its induction, but also on the specific cell population undergoing ferroptotic cell death [83].

#### 3.3.3. Stromal Crosstalk

Cancer-associated fibroblasts (CAFs) and tumor-associated mesenchymal stromal/stem cells (TA-MSCs) represent the main stromal components of the TME which contribute to tumor development by paracrine signaling, extracellular vesicle transfer, cell–cell interactions, extracellular matrix remodeling, immunosuppression, and therapy resistance [89,90]. CAFs can originate from different cellular sources, including resident fibroblasts, bone marrow-derived fibrocytes, MSCs, endothelial cells, epithelial cells, pericytes, smooth muscle cells, and adipocytes [89]. Similarly, TA-MSCs have been identified in several primary tumors and metastatic sites and can promote tumor growth, metastasis, stemness, chemoresistance, angiogenesis, and immune suppression by interacting with tumor cells and other stromal components [90].

CAFs can modulate ferroptosis in cancer cells. Particularly, it has been reported that CAFs release different factors which inhibit ferroptosis and contribute to tumor growth and therapy resistance in several tumor models. For example, in gastric cancer, CAF-derived exosomal miR-522 suppresses ferroptosis by targeting ALOX15, thus determining a decrease in lipid peroxidation and contributing to chemoresistance [91]. In pancreatic cancer, CAF-derived exosomal miR-3173-5p alters ACSL4 function, determining a reduction in ferroptotic lipid peroxidation and developing resistance to gemcitabine [92]. In addition, other CAF-secreted factors can promote the activation of HSF1-dependent pathways in cancer cells. In adult glioblastoma models, this mechanism determined an upregulation of SLC7A11 and inhibition of ferroptosis [93].

However, CAFs do not exhibit only anti-ferroptotic effects. Indeed, it is reported that CAFs can also increase tumor cells’ susceptibility to ferroptosis. In gastric cancer, CAF-derived exosomal DACT3-AS1 downregulates GPX4 in oxaliplatin-treated cells, thereby promoting ferroptosis and increasing oxaliplatin sensitivity [94]. These findings indicate that CAFs exhibit different behavior in modulation of ferroptosis, which depends on the tumor type, the CAF-secreted factors and the therapeutic intervention [89].

TA-MSCs play an important role in stromal communication since they can migrate to tumor sites, become incorporated into tumor stroma, and promote tumor progression by releasing cytokines, and participating in immune modulation, angiogenesis, and metastasis [90]. Evidence from pediatric neuroblastoma provides further insight into the relationship between tumor-associated MSCs and ferroptosis. Neuroblastoma-associated bone marrow MSCs (NB-BMSCs) promoted tumor cell growth and migration and exhibited increased resistance to several chemotherapeutic drugs. Interestingly, these stromal cells were more susceptible to ferroptosis than normal BMSCs, showing impairment of the Xc^-^-GSH-GPX4 pathway and increased sensitivity to the ferroptosis inducers erastin and RSL3 [31]. Moreover, BMSCs from neuroblastoma patients with bone marrow metastasis showed lower SLC7A11 and GPX4 expression than those from patients without bone marrow metastasis, indicating a reduced anti-ferroptotic capacity [31]. Ferroptosis susceptibility was also observed in CAF-MSCs isolated from neuroblastoma tumors, suggesting that ferroptosis may represent a potential strategy to target tumor-supportive stromal cells within the neuroblastoma microenvironment [31].

## 4. Ferroptosis and Therapy Resistance

Therapy resistance is one of the main causes of cancer treatment failure, tumor recurrence, and tumor progression. Ferroptosis is closely involved in therapy resistance, since cancer cells can avoid therapy-induced oxidative stress by promoting activation of anti-ferroptotic defense mechanisms and inhibiting lipid peroxidation [95,96]. Particularly, in tumors resistant to anti-cancer treatment, molecules involved in ferroptosis are down-regulated, while anti-ferroptotic compounds are up-regulated, thereby promoting survival of cancer cells [96].

There is a close link between ferroptosis resistance and chemoresistance, the inhibition of ferroptosis in tumor cells contributes to treatment failure. After chemotherapy the inhibition of ferroptosis is guaranteed by the activation of the SLC7A11-GSH-GPX4 axis which determines a reduction in lipid peroxide production [95,96]. Interestingly, GPX4 is an important modulator of chemoresistance, its activation induces chemoresistance, while its inhibition can restore ferroptosis and enhance the responses to chemotherapy [96]. This relationship has also been investigated in pediatric neuroblastoma, where ferroptosis susceptibility appears to be closely linked to chemoresistance. In patient-derived xenograft (PDX) models and PDX-derived organoids of high-risk neuroblastoma, Mañas et al. demonstrated an enrichment of ferroptosis-related antioxidant pathways in refractory tumors and highlighted that chemotherapy can interfere with specific ferroptosis-inducing mechanisms. In particular, chemotherapy-induced GPX4 upregulation antagonized the activity of GPX4-targeting ferroptosis inducers, indicating that the efficacy of ferroptosis-based combinations may depend on their mechanism of action [26].

Further evidence has been observed in neuroblastoma-derived models. In the multidrug-resistant HTLA-ER model, derived from the MYCN-amplified stage IV HTLA-230 neuroblastoma cell line, pharmacological inhibition of BMI-1 by PTC596 reduced intracellular GSH levels, increased peroxide production and lipid peroxidation, and induced ferroptotic cell death [32]. These findings further support ferroptosis induction as a potential strategy to overcome drug resistance in neuroblastoma.

By comparison, in adult tumor models, RelB-mediated GPX4 upregulation contributes to tamoxifen resistance in breast cancer, while albumin-bound paclitaxel sensitizes glioblastoma cells to temozolomide by downregulating GPX4 and promoting ferroptosis [94,96,97].

NRF2-dependent defense mechanisms have a key role in therapy resistance by stimulating the expression of several anti-ferroptotic genes, including SLC7A11, GPX4, FSP1, ABCC5, MT1G, and SOD2 [95]. It has been demonstrated that in different tumor models the activation of NRF2 induces resistance to several drugs, such as sorafenib, cisplatin, and temozolomide, while the inhibition of NRF2-related pathways promotes ferroptosis activation and improves therapeutic response [95,96]. This mechanism is also relevant to pediatric-derived osteosarcoma models. In cisplatin-resistant MG-63/DDP and Saos-2/DDP cells, increased activation of the STAT3/NRF2/GPX4 axis was associated with reduced ferroptosis susceptibility. Treatment with the ferroptosis inducers erastin or RSL3, as well as STAT3 inhibition, promoted ferroptosis and enhanced cisplatin sensitivity [35]. In adult tumor models, NRF2 inhibition reverses resistance to GPX4 inhibitor-induced ferroptosis in head and neck cancer, while ATF3 increases cisplatin sensitivity in gastric cancer by blocking NRF2/SLC7A11 signaling [98,99].

Recent evidence in B-cell acute lymphoblastic leukemia (B-ALL) further supports the relationship between ferroptosis and treatment response. Garcia-Gimenez et al. demonstrated that CREBBP loss-of-function sensitizes B-ALL cells to BCL2 inhibition with venetoclax through ferroptotic cell death. In isogenic CREBBP-mutant models, venetoclax increased lipid peroxidation, while Liproxstatin-1 significantly rescued cell viability, providing functional evidence of ferroptosis. Genetic and pharmacological CREBBP inactivation increased sensitivity to BCL2 inhibition, and this therapeutic interaction was also validated in vivo [36].

Impairment of iron and lipid metabolism influence ferroptosis susceptibility thus causing therapy resistance. Decreased levels of intracellular iron can avoid ferroptosis initiation and promote chemoresistance, instead biological processes which determines an increase in LIP may promote ferroptotic cell death [96]. In colorectal cancer, inhibition of the KLF5/LIF/MTF1/FPN1 axis induces iron overload and sensitizes cancer cells to oxaliplatin, while inhibition of glutaredoxin 5 or AEBP1 increases free iron levels and reverses cisplatin resistance in head and neck or oral cancer models [100,101,102]. Similarly, lipid remodeling regulates therapy response: ACSL4 promotes ferroptosis execution, and modulation of ACSL4-mediated ferroptosis can overcome oxaliplatin resistance in colorectal cancer [96,103]. Conversely, SCD1-driven MUFA production contributes to ferroptosis resistance and tumor recurrence, suggesting that targeting lipid remodeling may improve treatment efficacy [104].

The role of lipid remodeling in ferroptosis resistance has also been demonstrated in high-risk neuroblastoma models, including pediatric-derived cell lines. It has been reported that SCD1-mediated MUFA synthesis is involved in ferroptosis resistance, highlighting that genetic or pharmacological SCD1 inhibition is able to restore ferroptosis sensitivity in resistant neuroblastoma cells. Moreover, arachidonic acid supplementation further promoted ferroptotic cell death by increasing the PUFA/MUFA ratio [33].

Ferroptosis also influences radiotherapy and immunotherapy resistance. Radiotherapy can induce oxidative stress and lipid peroxidation, thereby promoting ferroptotic cell death; however, cancer cells may acquire radioresistance by enhancing anti-ferroptotic defenses [9,96].

The relationship between ferroptosis and radiotherapy has also been demonstrated in pediatric-derived medulloblastoma models. In particular, it has been reported that in SHH medulloblastoma FANCD2 is responsible for radioresistance by limiting ferroptosis. FANCD2 deficiency determined an increase in intracellular Fe^2+^ accumulation and an impairment of GPX4 activity, thus contributing to ferroptosis susceptibility and enhancing radiosensitivity. In addition, FANCD2 silencing together with radiotherapy significantly inhibited tumor growth in an orthotopic mouse model [25].

However, the combination of ferroptosis induction with radiotherapy also raises important safety considerations, particularly in pediatric CNS tumors. Although ferroptosis induction may enhance tumor radiosensitivity, ferroptosis has also been implicated in radiation-induced injury to normal brain tissue [105]. This issue may be particularly relevant in pediatric patients, since radiotherapy for pediatric CNS tumors can be associated with long-term neurological sequelae, including neurocognitive deficits, endocrine dysfunctions, and cerebrovascular complications [106].

For example, the CoQ-FSP1 axis drive ferroptosis and radiation resistance in KEAP1-inactive lung cancers, and upregulation of CoQ can shift ferroptosis dependence from GPX4 to FSP1 in acquired radioresistance [63,107]. Conversely, promoting ferroptosis can enhance radiosensitivity, as shown by studies targeting SLC7A11, GPX4, or Cu–Fe balance-related pathways [9,96].

Finally, ferroptosis can influence immunotherapy resistance. CD8+ T cells can promote tumor ferroptosis through IFN-γ-mediated suppression of system Xc^-^, thereby contributing to the efficacy of immune checkpoint blockade [63]. However, tumors may evade immunotherapy by limiting ferroptosis. For instance, TYRO3 induces resistance to anti-PD-1/PD-L1 therapy by limiting innate immunity and tumoral ferroptosis, whereas BET inhibition can potentiate melanoma ferroptosis and improve immunotherapy response through AKR1C2 inhibition [108,109].

Importantly, alterations in the expression of ferroptosis-related molecules should not alone be interpreted as evidence that ferroptosis causally determines treatment resistance. Stronger mechanistic evidence is supported by functional validation, including genetic manipulation of ferroptosis regulators and/or rescue of treatment-induced cell death by ferroptosis inhibitors.

**Figure 3:**
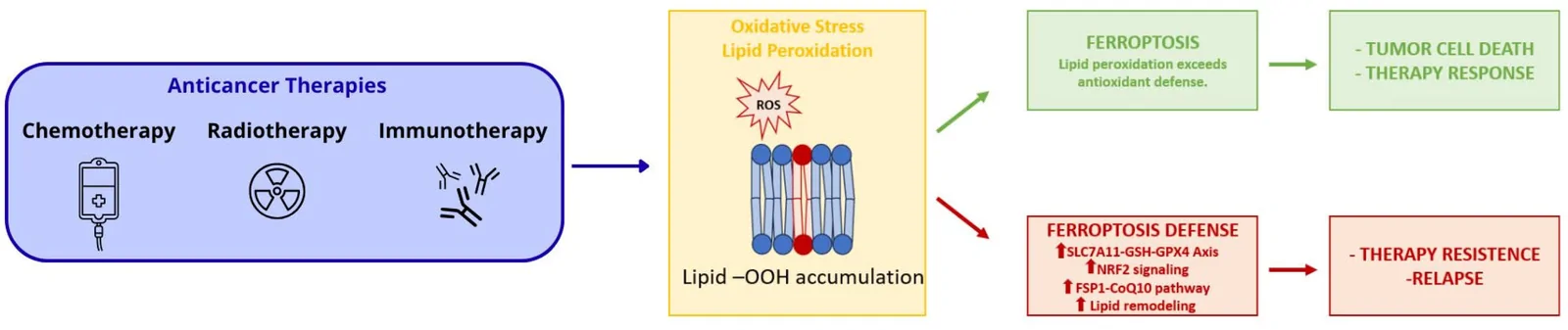
Ferroptosis as a determinant of cancer therapy response and resistance. Anticancer therapies, including chemotherapy, radiotherapy, and immunotherapy, can promote oxidative stress, lipid peroxidation, and the accumulation of lipid hydroperoxides (Lipid-OOH). When lipid peroxidation exceeds cellular antioxidant defenses, ferroptosis contributes to tumor cell death and therapeutic response. Conversely, activation of ferroptosis defense mechanisms, including the SLC7A11-GSH-GPX4 axis, NRF2 signaling, the FSP1-CoQ10 pathway, and adaptive lipid remodeling, limits ferroptotic cell death and promotes therapy resistance and disease relapse. Green arrows indicate the pathway leading to ferroptosis and therapeutic response, red arrows indicate the activation of ferroptosis defense mechanisms associated with therapy resistance, and the blue arrow indicates therapy-induced oxidative stress and lipid peroxidation. Abbreviations: SLC7A11, solute carrier family 7 member 11; GSH, glutathione; GPX4, glutathione peroxidase 4; NRF2, nuclear factor erythroid 2-related factor 2; FSP1, ferroptosis suppressor protein 1; CoQ10, coenzyme Q10.

Therefore, targeting ferroptosis-related pathways may represent a promising strategy to overcome resistance to chemotherapy, radiotherapy, and immunotherapy, although the heterogeneity of tumor metabolism and microenvironmental context must be carefully considered [9,96] (Figure 3).

## 5. Therapeutic Targeting of Ferroptosis

The increasing understanding of the molecular mechanisms regulating ferroptosis has opened new therapeutic opportunities in cancer treatment. Cancer cells can develop anti-ferroptotic defense systems to avoid oxidative and metabolic stress [95,110,111]. Therefore, pharmacological induction of ferroptosis represents a promising strategy to directly induce cancer cells death and overcome resistance to conventional anticancer therapies. Different ferroptosis-inducing agents (FINs) have been developed to target key compounds of ferroptosis defense mechanisms, particularly System Xc^-^ and the GSH-GPX4 axis [9,10,112]. Moreover, increasing evidence indicates that ferroptosis induction may enhance the efficacy of conventional anticancer treatments, supporting the development of combination therapeutic strategies [9,84,113].

### 5.1. Ferroptosis Inducers

Ferroptosis inducers target biological mechanisms which are responsible for redox homeostasis maintenance by inducing cancer cell death. Erastin and RSL3 represent two of the principal ferroptosis inducers which act in different manner: erastin indirectly impairs GPX4 activity by targeting System Xc^-^, whereas RSL3 directly inhibits GPX4 [9,10,112].

Erastin is a classical ferroptosis inducer that inhibits System Xc^-^, thereby reducing cystine uptake [9,10]. The consequent reduction in intracellular cysteine availability limits GSH synthesis and indirectly impairs GPX4 activity, leading to lipid peroxide accumulation and ferroptotic cell death [9,10]. Although erastin has shown anticancer activity in experimental models, its poor water solubility and unfavorable pharmacological properties limit its potential clinical application. To overcome these limitations, more stable derivatives, such as imidazole ketone erastin (IKE), have been developed and have shown improved pharmacological properties and antitumor activity in preclinical models [9,95,114].

The direct inhibition of GPX4 represent another important therapeutic strategy. In particular, RSL3 covalently binds to GPX4 and inhibits the activation of its enzymatic activity, inhibiting lipid hydroperoxide elimination and consequently contributing to their lethal accumulation [9,112]. In experimental cancer models, other direct GPX4 inhibitors (ML162 and ML210) enhance ferroptosis in cancer cells [9,115,116]. Moreover, other ferroptosis inducers are able to drive degradation of GPX4; among these compounds, FIN56 degrades GPX4 and simultaneously reduces the availability of coenzyme Q10, thus increasing cancer cell susceptibility to ferroptosis [9,117].

Beyond these conventional ferroptosis inducers, an increasing number of small molecules and natural compounds have been proposed ed as ferroptosis-inducing agents in cancer [118]. Interestingly, several bioactive compounds derived from traditional Chinese medicine have also emerged as potential modulators of ferroptosis through mechanisms involving amino acid, iron, and lipid metabolism [119]. Dihydroartemisinin has been shown to induce lipid peroxidation and ferroptosis in experimental lung cancer models [120], while artesunate promoted ferroptosis in osteosarcoma through NCOA4-mediated ferritinophagy, iron accumulation, and impairment of the xCT/GPX4 antioxidant pathway [121]. In addition, baicalein induced ferroptosis in colorectal cancer models through inhibition of the JAK2/STAT3/GPX4 axis [122]. Although these compounds broaden the spectrum of potential ferroptosis-targeting agents, most available evidence remains preclinical, and further studies are required to establish their pharmacological properties, tumor selectivity, safety, and potential applicability to pediatric cancers.

The therapeutic potential of ferroptosis induction has also been demonstrated in pediatric neuroblastoma models. In MYCN-amplified neuroblastoma, increased iron uptake and ROS production create a metabolic dependency on the System Xc^-^-GSH antioxidant pathway. Accordingly, pharmacological inhibition of this pathway promoted ferroptotic cell death, while ferroptosis inhibition with ferrostatin-1 or iron chelation reduced treatment-induced cytotoxicity [27]. Therefore, MYCN-driven metabolic features may represent a specific vulnerability to ferroptosis-inducing strategies in neuroblastoma.

### 5.2. Combination Therapeutic Strategies

It is very interesting and relevant to consider the possibility to use ferroptosis inducers in combination with conventional anticancer therapies.

Indeed, cancer cells characterized by resistance to anti-cancer therapies frequently develop adaptive mechanisms which counteract ferroptosis initiation [95,110,111]. Therefore, pharmacological stimulation of ferroptosis can induce death of resistant cancer cells and restore therapeutic sensitivity.

#### 5.2.1. Combination with Chemotherapy

Firstly, the combination of ferroptosis inducers with chemotherapy represents a promising strategy to prevent drug resistance and ameliorate treatment efficacy. Chemotherapeutic drugs are responsible for the development of oxidative stress; however, cancer cells are able to counteract this stress by devising adaptive mechanisms that inhibit ferroptosis. Therefore, the development of therapeutic approaches aimed to target these adaptative mechanisms can promote therapy-induced lipid peroxidation and induce ferroptotic cell death [7,96].

In this context, the main therapeutic target is represented by SLC7A11-GSH-GPX4 axis. Increased GPX4 activity can protect cancer cells against therapy-induced oxidative damage, whereas inhibition of this pathway can promote ferroptotic cell death and restore treatment sensitivity [7,96]. The therapeutic potential of combining ferroptosis induction with chemotherapy has also been demonstrated in high-risk neuroblastoma. In chemoresistant neuroblastoma PDX models, the combination of auranofin with conventional chemotherapy enhanced antitumor activity compared with chemotherapy alone and prolonged survival [26]. Mechanistically, auranofin promoted ferritinophagy and lysosomal iron accumulation, thereby increasing ferroptosis susceptibility. These findings support the potential of ferroptosis-inducing strategies to enhance chemotherapy efficacy in resistant neuroblastoma [26].

Similar mechanisms have been described in other tumor models. In breast cancer cells, RelB-mediated GPX4 upregulation inhibits ferroptosis and contributes to tamoxifen resistance, suggesting GPX4 as therapeutic target to induce ferroptosis thus restoring treatment sensitivity [96,97]. Moreover, inhibition of CDK1 can overcome oxaliplatin resistance in colorectal cancer by regulating ACSL4-mediated ferroptosis [96,103]. This evidence reveals that ferroptosis induction may promote the efficacy of anticancer treatments and overcome acquired resistance by targeting specific ferroptosis defense mechanisms.

#### 5.2.2. Combination with Immunotherapy

Since there is a close interaction between ferroptosis and antitumor immunity, the combination of ferroptosis and immunotherapy could be considered a novel promising therapeutic approach. As already discussed, activated CD8+ T cells release IFN-γ, which reduces the expression of System Xc^-^ components in cancer cells, thus decreasing cystine uptake and increasing lipid peroxidation and ferroptotic cell death [9,84]. Therefore, ferroptosis contributes to the antitumor activity of immune checkpoint blockade.

Interestingly, it has been demonstrated a potential therapeutic interaction between ferroptosis and immune-mediated cytotoxicity in pediatric-derived neuroblastoma cell models. Erastin-induced ferroptosis determined an increase in the expression of ULBP1—a ligand of the activating NK-cell receptor NKG2D—via an ATF4-dependent mechanism. Moreover, treatment with erastin promoted the susceptibility of neuroblastoma cells to NK cell-mediated cytotoxicity. Conversely, this effect was reduced by the ferroptosis inhibitor Liproxstatin-1 [34]. Ultimately, ferroptosis induction may represent a potential strategy to enhance NK cell-mediated antitumor activity in neuroblastoma.

The relationship between ferroptosis and immunotherapy is further supported by evidence showing that radiotherapy and immunotherapy can cooperate to promote tumor lipid oxidation and ferroptosis through the repression of SLC7A11 [7,9,113]. Moreover, ferroptosis-related mechanisms can influence resistance to immune checkpoint inhibitors. TYRO3 induces resistance to anti-PD-1/PD-L1 therapy by limiting innate immunity and tumoral ferroptosis, whereas inhibition of TYRO3 restores ferroptosis and sensitizes resistant tumors to anti-PD-1 therapy [9,108]. Conversely, BET inhibition can promote melanoma ferroptosis and improve immunotherapy efficacy through AKR1C2 inhibition [9,109].

However, the interaction between ferroptosis and antitumor immunity is complex. Ferroptosis may promote tumor elimination and enhance antitumor immune responses, but excessive lipid peroxidation can also damage immune cells and impair their antitumor functions. Therefore, the therapeutic effects of combining ferroptosis inducers with immunotherapy depend on the cellular and metabolic characteristics of the tumor microenvironment [9].

### 5.3. Clinical Potential of Ferroptosis-Based Therapies

Although preclinical studies have so far yielded promising results, the clinical translation of therapies based on the use of ferroptosis and its components as targets remains one of the greatest and most exciting challenges in the medical and biological field. However, the main inducers of ferroptosis (Erastin, RSL3, ML162, and ML210) are still experimental compounds that are not yet used in clinical practice. Their limited use is due to several intrinsic characteristics, including poor water solubility, unfavorable pharmacokinetic properties, limited tumor selectivity, and potential toxicity to normal tissues [7,9,95].

Another relevant factor which influences the clinical translation of ferroptosis-based therapies is the tumor heterogeneity. In particular, cancer cells differ considerably in their iron metabolism, membrane lipid composition, antioxidant capacity, and dependence on ferroptosis defense pathways. Consequently, sensitivity to ferroptosis induction may vary among different tumor types and even among distinct cancer cell populations within the same tumor. The identification of reliable biomarkers able to predict ferroptosis sensitivity will therefore be essential for selecting patients who may benefit from ferroptosis-based therapeutic approaches [7,9].

In this context, the identification of biomarkers for minimally invasive assessment and longitudinal disease monitoring represents an important future direction. Several ferroptosis-associated biomarkers have been proposed, including lipid peroxidation products such as malondialdehyde (MDA) and 4-hydroxynonenal (4-HNE), alterations in iron metabolism, and changes in ferroptosis-related genes and proteins, including SLC7A11, ACSL4, and TFRC [123]. In pediatric neuroblastoma, ferroptosis-related gene signatures have been associated with clinical outcome, supporting their potential value for patient stratification [124]. Circulating lipid peroxidation products and other ferroptosis-associated molecules detectable in body fluids may represent potential candidates for future monitoring strategies, but their specificity and clinical utility for assessing ferroptosis in pediatric cancer remain to be established [125]. Therefore, prospective studies are required to determine whether minimally invasive ferroptosis-associated biomarkers could be used for patient selection and longitudinal monitoring during ferroptosis-targeting therapies. Furthermore, ferroptosis is not exclusively restricted to cancer cells. Its induction may also affect immune and stromal cells within the TME and potentially damage normal tissues. Therefore, the development of tumor-selective delivery systems represents an important challenge for clinical translation.

In pediatric brain tumors, therapeutic delivery represents an additional challenge because the blood–brain barrier (BBB) and the tumor-associated blood-tumor barrier (BTB) can limit drug penetration and the achievement of effective intratumoral drug concentrations [126,127]. Moreover, barrier permeability is highly heterogeneous across different pediatric brain tumor types, molecular subgroups, anatomical locations, and even among different regions within the same tumor, further supporting the need for tumor-specific delivery strategies [127].

Nanoparticles, hydrogels, and liposomes are currently being investigated to improve the selective delivery of ferroptosis inducers to cancer cells while limiting toxicity to normal tissues [7,9].

**Figure 4:**
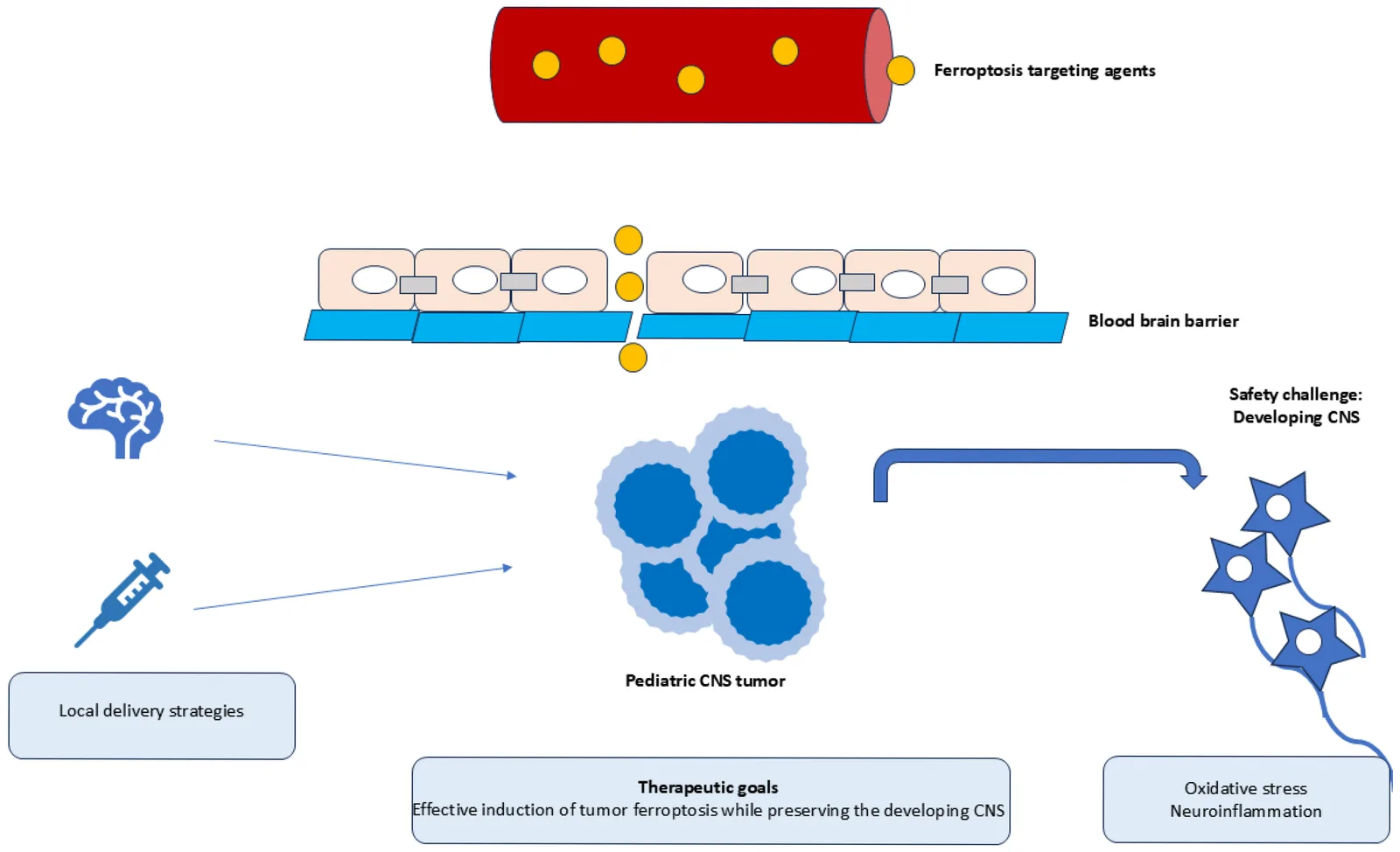
Translational challenges and potential delivery strategies for ferroptosis-targeting therapies in pediatric CNS tumors. The blood–brain barrier may limit the penetration of systemically administered ferroptosis-targeting agents and their exposure at the tumor site. Local delivery strategies may represent potential approaches to overcome this limitation. However, ferroptosis induction may also affect normal cells of the developing CNS, potentially contributing to oxidative stress and neuroinflammation. Therefore, effective tumor ferroptosis induction should be balanced with preservation of the developing CNS. CNS, central nervous system.

In pediatric CNS tumors, several strategies are being investigated to overcome the limitations imposed by the BBB and improve drug delivery to the tumor. These include convection-enhanced delivery (CED), which allows for direct intraparenchymal administration of therapeutic agents, and intrathecal and intraventricular administration into the cerebrospinal fluid [128,129]. Local delivery approaches based on biodegradable polymers or injectable hydrogels placed within the surgical resection cavity have also been investigated as strategies to bypass the BBB and increase drug exposure at the tumor site [129]. However, the applicability of these approaches depends on tumor location, dissemination pattern, and surgical accessibility [129]. Although these delivery strategies have not yet been specifically established for ferroptosis-inducing agents in pediatric CNS tumors, they may represent potential platforms for the future development of ferroptosis-based therapies. These major translational challenges and potential delivery strategies for ferroptosis-targeting therapies in pediatric CNS tumors are summarized in Figure 4.

An additional concern for the clinical translation of ferroptosis-based therapies is their potential toxicity to normal CNS tissues. The brain may be particularly vulnerable to ferroptosis because of its high lipid and iron content and high oxygen consumption, and ferroptosis has been extensively implicated in neurodegeneration and cerebral ischemia [130]. This issue deserves particular consideration in pediatric patients, as cancer and its treatments may affect the developing brain and result in long-term neurological and neurocognitive sequelae [131]. Therefore, the potential effects of ferroptosis induction on normal neural cells and developing tissues should be carefully investigated to define a safe therapeutic window before the clinical application of ferroptosis-inducing strategies in pediatric CNS tumors.

Combination strategies may represent one of the most promising approaches for the future clinical application of ferroptosis-based therapies. Rather than using ferroptosis inducers as monotherapies, their combination with chemotherapy or immunotherapy may increase therapeutic efficacy and overcome treatment resistance [7,9,95]. Nevertheless, further studies are required to define optimal therapeutic combinations, identify predictive biomarkers, improve the pharmacological properties and tumor selectivity of ferroptosis inducers, and determine their safety in patients. Overall, although ferroptosis represents a promising therapeutic vulnerability in cancer, its successful clinical translation will require a better understanding of tumor-specific ferroptosis susceptibility and of the complex interactions between ferroptotic cell death and the TME [7,9]. The main ferroptosis-targeting agents and combination therapeutic strategies, together with their mechanisms, potential applications, and major translational limitations, are summarized in Table 2.

## 6. Discussion

Ferroptosis is a particular form of regulated cell death with a key role in cancer biology [1,2,3,4,7]. It is induced by iron-dependent membrane lipid peroxidation and is regulated by iron metabolism, membrane lipid composition, cellular metabolism, and multiple antioxidant defense systems [1,2,11,37,42]. Cancer cells exhibit characteristic features, such as increased iron demand, high oxidative stress, and impaired lipid metabolism. These metabolic events can promote tumor progression but—at the same time—promote susceptibility to ferroptosis [3,4,7,55]. Therefore, ferroptosis has emerged as a promising therapeutic target to counteract cancer development and progression [1,2,3,4,7].

Moreover, metabolic reprogramming, CSCs plasticity, and the resistance to oxidative stress of disseminating tumor cells can modify ferroptosis susceptibility, highlighting the relationship between this form of cell death and tumor progression, recurrence, and metastatic dissemination [65,66,67,68,69,70,71,72,73,74,75,76,77,78,79,80,132,133].

The role of ferroptosis within the TME is particularly complex and context-dependent. Importantly, in cancer cells ferroptosis may promote antitumor immunity, whereas in antitumor immune cells it may instead impair immune surveillance and enhance tumor immune evasion [83,84,85,86,87,88]. This context-dependent and cell-specific activity represents an important challenge for the therapeutic modulation of ferroptosis and highlights the need to determine which cellular populations should be selectively targeted or protected.

The close relationship between ferroptosis and therapy resistance further emphasizes its therapeutic relevance. Conversely, targeting these pathways can restore ferroptosis susceptibility and improve therapeutic response, supporting the use of ferroptosis induction as a strategy to overcome treatment resistance [9,95,96]. However, the relationship between ferroptosis and treatment response appears to be strongly dependent on tumor type, molecular background, and therapeutic context. Therefore, ferroptosis induction should not be considered a universally applicable strategy to overcome treatment resistance, and the molecular determinants of response need to be defined for individual tumor types.

Moreover, combining ferroptosis-inducing agents with conventional anti-cancer therapies may promote treatment efficacy and selectively eliminate cancer cell population which is resistant to conventional treatments [89,90,100]. Nevertheless, the majority of ferroptosis inducers remains experimental compounds, and their clinical translation is restricted by poor pharmacological properties, lack of tumor selectivity, potential toxicity to normal tissues, and the heterogeneity of ferroptosis susceptibility among and within tumors [7,9]. These limitations suggest that the clinical development of ferroptosis-based therapies will probably require molecularly selected patients and rational combination strategies rather than the indiscriminate use of ferroptosis inducers across different malignancies.

These aspects are particularly relevant in pediatric oncology. Pediatric cancers are biologically different from adult ones in terms of developmental origin, genomic and epigenetic landscape, and metabolic characteristics [18,19,20]. Therefore, mechanisms identified in adult cancer models may not necessarily be directly translated to pediatric malignancies. Furthermore, although emerging evidence supports a role for ferroptosis in pediatric cancer, current knowledge remains concentrated in specific tumors, particularly neuroblastoma, with additional evidence emerging in acute lymphoblastic leukemia, osteosarcoma, and medulloblastoma [16,17,23,24,25]. Existing preclinical studies suggest that targeting ferroptosis may enhance therapeutic efficacy and overcome treatment resistance in pediatric cancer models [17,23,26]. However, the limited evidence in pediatric malignancies represents an important knowledge gap and emphasizes the need to investigate ferroptosis within individual tumor entities and molecular subgroups rather than considering pediatric cancers as a homogeneous group.

## 7. Conclusions

Future studies can define the tumor-specific molecular drivers of ferroptosis susceptibility in pediatric cancers and identify biomarkers to predict and to monitor treatment response [7,74]. Particular attention should also be devoted to the effects of ferroptosis induction on normal developing tissues and on the immune and stromal compartments of the pediatric TME, as these aspects will be necessary to define safe therapeutic windows [7,81]. The development of more selective ferroptosis inducers and tumor-targeted delivery systems may contribute to improve therapeutic efficacy, limiting off-target toxicity [7,9].

Overall, ferroptosis represents a promising but still incompletely explored field in pediatric cancer. A deeper understanding of its molecular regulation, its interaction with the TME, and its contribution to therapy resistance will be necessary to translate current preclinical findings into clinical practice [7,14,15]. Integrating ferroptosis-inducer treatment with existing anticancer therapies and personalizing these strategies to the specific biological characteristics of individual pediatric tumors may ultimately contribute to overcome treatment resistance, reducing disease recurrence, and improving therapeutic outcomes in children with cancer [14,15,16,17,23,24,26].

## Figures and Tables

**Table 2:** Main therapeutic strategies to target ferroptosis in cancer. Overview of the main pharmacological agents and combination strategies used to induce ferroptosis, their molecular targets and mechanisms of action, potential therapeutic applications, and major limitations to clinical translation.

Strategy/Agent	Molecular Target	Mechanism of Ferroptosis Induction	Therapeutic Potential	Main Translational Limitation	Ref.
**Erastin**	System Xc^-^/SLC7A11	Inhibits cystine uptake, depletes GSH, and indirectly impairs GPX4 activity	Experimental induction of ferroptosis in cancer cells	Poor water solubility and unfavorable pharmacological properties	[9,10]
**Imidazole ketone erastin (IKE)**	System Xc^-^/SLC7A11	More stable erastin derivative that reduces cystine uptake and promotes lipid peroxidation	Improved antitumor activity in preclinical models	Still limited to preclinical investigation	[9,95,114]
**RSL3**	GPX4	Directly inhibits GPX4, preventing lipid hydroperoxide detoxification	Strong experimental induction of ferroptosis	Limited pharmacokinetic properties and potential off-target toxicity	[9,112]
**ML162**	GPX4	Direct GPX4 inhibition and accumulation of lipid peroxides	Experimental targeting of GPX4-dependent cancer cells	Preclinical compound; limited clinical applicability	[9,116]
**ML210**	GPX4	Covalent GPX4 inhibition and lethal lipid peroxide accumulation	Selective targeting of ferroptosis-sensitive cancer cells	Limited bioavailability and preclinical status	[9,115]
**FIN56**	GPX4/CoQ10	Promotes GPX4 degradation and reduces CoQ10-dependent antioxidant protection	Simultaneous impairment of complementary ferroptosis defense mechanisms	Preclinical status and limited tumor selectivity	[9,117]
**Combination with chemotherapy**	Mainly SLC7A11-GSH-GPX4 and ACSL4-related pathways	Increases therapy-induced oxidative stress and lipid peroxidation	May restore sensitivity and overcome acquired drug resistance	Tumor heterogeneity and variable ferroptosis susceptibility	[7,96,97,103]
**Combination with immunotherapy**	IFN-γ-System Xc^-^ axis and tumor ferroptosis defense pathways	Promotes tumor-cell lipid peroxidation and may enhance immune checkpoint blockade	Potential improvement in antitumor immune responses	Context-dependent effects and possible ferroptosis of antitumor immune cells	[9,84,108,109]

## Data Availability

No new data were created or analyzed in this study. Data sharing is not applicable to this article.
